# Improving the Performance of Continuous-Variable Measurement-Device-Independent Quantum Key Distribution via a Noiseless Linear Amplifier

**DOI:** 10.3390/e23121691

**Published:** 2021-12-16

**Authors:** Fan Jing, Weiqi Liu, Lingzhi Kong, Chen He

**Affiliations:** College of Information Science and Technology, Northwest University, Xi’an 710127, China; jingfan1217@163.com (F.J.); konglingzhi@stumail.nwu.edu.cn (L.K.)

**Keywords:** CV-MDI-QKD, NLA, the performance

## Abstract

In the continuous variable measurement-device-independent quantum key distribution (CV-MDI-QKD) protocol, both Alice and Bob send quantum states to an untrusted third party, Charlie, for detection through the quantum channel. In this paper, we mainly study the performance of the CV-MDI-QKD system using the noiseless linear amplifier (NLA). The NLA is added to the output of the detector at Charlie’s side. The research results show that NLA can increase the communication distance and secret key rate of the CV-MDI-QKD protocol. Moreover, we find that the more powerful the improvement of the performance with the longer gain of NLA and the optimum gain is given under different conditions.

## 1. Introduction

With the development of photoelectric technology, many physical phenomena of quantum theory [1] have been verified through observation, which attracts many researchers to consider its application. Quantum key distribution (QKD) [2,3] is one of the applications, in which two trusted communication parties (Alice and Bob) are allowed to exchange the cryptographic key through a quantum channel at the existence of eavesdropping. Its theoretical unconditional security is guaranteed by the laws of Heisenberg’s uncertainty principle [4] and no-clone theory [5,6]. The QKD protocols are mainly divided into the following two categories: the discrete variable quantum key distribution (DVQKD) [7,8,9,10] and the continuous variable quantum key distribution (CVQKD) [11,12,13,14,15]. In theory, the unconditional security of QKD has been proven [16,17,18,19,20,21,22].

However, the deviation between the theoretical assumption and the actual implementation will effect the performance and may lead to a loophole in the practical system that could be used by Eve to intercept key information without being discovered. The loopholes involve the the laser source [23,24,25], the local oscillator [26,27], the beam splitter (BS) [28,29,30], the basis choice [31], and the detector [32,33,34,35,36,37]. Nowadays, researchers have proposed the CV-MDI-QKD protocol [38,39,40], which can defend all detector side channels. In the CV-MDI-QKD protocol, Alice and Bob each generate an Einstein–Podolsky–Rosen (EPR) state, and both of them send one mode of the EPR state to an untrusted third party, Charlie, for detection through the quantum channel. After the CV-MDI-QKD protocol was put forward, it was well analyzed in theory and demonstrated in experiments. However, the shortcomings of the communication distance and secret key rate of the CV-MDI-QKD system are still a problem.

In this paper, facing the above problem, the NLA is induced to improve the performance of the CV-MDI-QKD system. The amplification performance of the NLA is better because it can mechanically amplify the amplitude of the coherent state while retaining the excess noise at the initial level [41,42,43]. Thus, when only considering its successful runs, the NLA can compensate the effect of losses and could therefore be useful for quantum communication [44]. So, in theory, the NLA can greatly improve the signal-to-noise ratio of the transmission signal, so that the performance of the system can be improved. Here, we consider that we can add two NLAs to the output ports of two homodyne detectors on Charlie’s side. We find that the communication distance and secret key rate of the NLA-based CV-MDI-QKD system have been improved.

The structure of this paper is as follows. In Section 2, we first introduce the CV-MDI-QKD protocol, and then the CV-MDI-QKD protocol with NLA is investigated. Then, the performance of the CV-MDI-QKD protocol with NLA is analyzed in Section 3. Finally, we conclude the paper in Section 4.

## 2. The Scheme of CV-MDI-QKD with NLA

**Figure 1:**
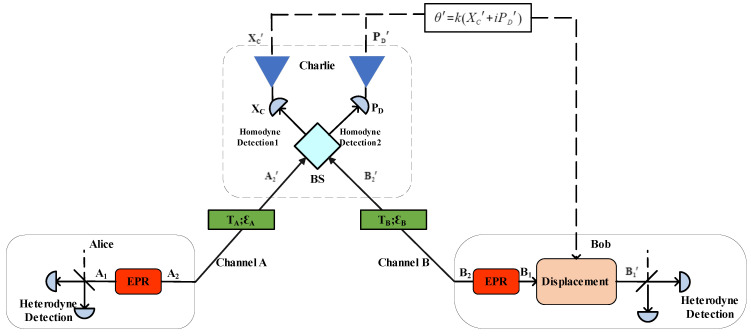
The EB scheme of the CV-MDI-QKD system with the noiseless linear amplifier. $T_{A}$ and $\epsilon_{A}$ are the transmittance and excess noise of channel A, respectively. $T_{B}$ and $\epsilon_{B}$ are the transmittance and excess noise of channel B, respectively. BS is the beam splitter with the splitting ratio 50:50.

The equivalent entanglement-based (E&B) model of the CV-MDI-QKD protocol is shown in Figure 1. Alice prepares an EPR state, keeps one of its mode $A_{1}$, and sends the other mode $A_{2}$ to Charlie by channel A. Bob also prepares another EPR state and keeps the mode $B_{1}$, and the mode $B_{2}$ is also sent to Charlie by channel B. Then, Charlie will obtain the quadratures $X_{C}$ and $P_{D}$ via homodyne detectors after interfering with the received modes $A_{2}^{\prime }$ and $B_{2}^{\prime }$ through a 50:50 BS. Then, $X_{C}$ and $P_{D}$ pass through their respective amplifiers. After being amplified by NLA, we record the measurement results $\left\{X_{C}^{\prime },P_{D}^{\prime }\right\}$, which will be announced by Charlie.

After Alice and Bob receive the measurement results announced by Charlie, Bob performs the displacement operation $D(\theta^{\prime })$ on the mode $B_{1}$ and obtains the mode $B_{1}^{\prime }$, where $\theta^{\prime }=k\left(X_{C}^{\prime }+iP_{D}^{\prime }\right)$ and *k* is the gain coefficient of the displacement operation. Then, Alice and Bob measure states $A_{1}$ and $B_{1}^{\prime }$, respectively, via heterodyne detector. Finally, they obtain the data $\left\{X_{A}^{\prime },P_{A}^{\prime }\right\}$, $\left\{X_{B}^{\prime },P_{B}^{\prime }\right\}$. If we assume that Bob’s EPR state preparation and displacement operation are untrusted, then the protocol could be seen as the well-known one-way CVQKD protocol using coherent states and heterodyne detection [45].

Moreover, due to the presence of Charlie in the CV-MDI-QKD system, there are two situations. Firstly, the symmetrical situation is that the distance $L_{AC}$ between Alice and Charlie is equal to the distance $L_{BC}$ between Bob and Charlie, i.e., $L_{AC}=L_{BC}$. In this case, the secure communication distance is relatively short due to the excess noise in the channel. Secondly, the asymmetrical case is that the third-party is infinitely close to Bob, that is, $L_{BC}=0$. In this case, the communication distance is significantly higher than the symmetrical case. Therefore, in this paper, we only consider the secret key rate in the asymmetric case.

**Figure 2:**
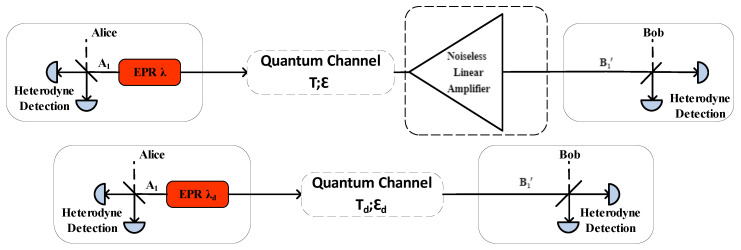
The equivalent method is used to analyze the CV-MDI-QKD system with NLA.

Assuming that there is such an equivalent in Figure 2, i.e., the modulation variance of the CV-MDI-QKD system without NLA is $V\left(\lambda \right)=\frac{1+\lambda^{2}}{1-\lambda^{2}}$, the channel transmittance is T, and the channel excess noise ϵ can be equivalent to the modulation variance of the NLA-based CV-MDI-QKD system, which is $V\left(\lambda_{d}\right)=\frac{1+\lambda_{d}^{2}}{1-\lambda_{d}^{2}}$, and the channel transmittance and excess noise are $T_{d}$ and $\epsilon_{d}$, respectively. Considering a thermal state ${\hat{\rho }}_{th}\left(\lambda_{ch}\right)=\left(1-\lambda_{ch}^{2}\right)\sum {}_{n=0}^{\infty }\lambda_{ch}^{2n}|n\rangle \langle n|$ with variance $\frac{1+\lambda_{ch}^{2}}{1-\lambda_{ch}^{2}}$ and the displacement $\beta =\beta_{x}+i\beta_{y}$, the displaced input thermal state can be given by:

(1) $$\hat{\rho }=\hat{D}\left(\beta \right){\hat{\rho }}_{th}\left(\lambda_{ch}\right)\hat{D}\left(-\beta \right)$$

When the input thermal state is amplified successfully by NLA, the thermal state $\hat{\rho }$ will be transformed into:

(2) $$\hat{\rho^{\prime }}=\hat{D}\left(g^{\prime }\beta \right){\hat{\rho }}_{th}\left(g\lambda_{ch}\right)\hat{D}\left(-g^{\prime }\beta \right)$$

where the variance is $\frac{1+g^{2}\lambda_{ch}^{2}}{1-g^{2}\lambda_{ch}^{2}}$. Here, $g^{\prime }$ is $g\frac{1-\lambda_{ch}^{2}}{1-{\left(g\lambda_{ch}\right)}^{2}}$, we set the parameter *g* to satisfy $g\lambda_{ch}<1$, $\lambda_{ch}$ is the compressibility of the incident thermal state, and the displacement of the thermal state is $g^{\prime }\beta$ after the amplification of NLA. The related parameters are given by:

(3) $$\begin{array}{cc} \; & \lambda_{d}=\lambda \sqrt{\frac{\left(g^{2}-1\right)\left(\epsilon -2\right)T-2}{(g^{2}-1)\epsilon T-2}}\\ \; & T_{d}=\frac{g^{2}T}{\left(g^{2}-1\right)T\left[\frac{1}{4}\left(g^{2}-1\right)\left(\epsilon -2\right)\epsilon T-\epsilon +1\right]}\\ \; & \epsilon_{d}=\epsilon -\frac{1}{2}\left(g^{2}-1\right)\left(\epsilon -2\right)\epsilon T \end{array}$$

where *g* is the amplification gain of NLA. The amplification effect of NLA on the system is essential to amplify the quantum state transmitted in the channel. As we all know, when the coherent state transmits in the channel, it will be interfered by the excess noise of the channel. Then, it will be regarded as a displacement thermal state. The NLA’s effect on this displaced thermal state can be described by Equation (3) when it is successfully amplified. However, the above equation only considers the amplification effect of the quantum state transmitted in the channel when the NLA amplification is successfully amplified. The successful amplification of NLA is actually probabilistic. Therefore, we must also consider the impact of NLA probabilistic amplification on the EPR state [45].

In general, the thermal state ρ can be regarded as the superposition of countless coherent states and can be expressed $\hat{\rho }=\int P\left(\alpha \right)|\alpha \rangle \langle \alpha |d\alpha$. The equation can be used in the EB model, and P(α) represents the probability of the coherent state $|\alpha \rangle$ being transmitted to Bob through the channel. If we selectively amplify the coherent state $|\alpha \rangle$ in the channel and only retain the successfully amplified state, that is, the non-uniform shielding of the EPR state, we will obtain a new thermal state finally. The corresponding entanglement parameter $\lambda_{d}$ is given as follows:

(4) $$\lambda_{d}=\frac{\lambda }{\sqrt{1-T\lambda^{2}\left(g^{2}-1\right)}}$$

Furthermore, only considering the case when the coherent state is successfully amplified by NLA, the other two equivalent parameters $T_{d}$ and $\epsilon_{d}$ will be recalculated. The EPR state is always prepared by Alice, and Alice obtains the result of the heterodyne detection $\alpha_{A}$ in one mode $|\lambda \rangle$ of the EPR state, the amplitude of which is proportional to $\lambda \alpha_{A}$. This coherent state is sent to Bob through a quantum channel with a transmittance *T*, which transforms its amplitude to $\propto \sqrt{T}\lambda \alpha_{A}$. The displacement thermal state β can thus be taken as:

(5) $$\beta =\sqrt{T}\lambda \alpha_{A}$$

After being amplified by the NLA, the displacement thermal state β becomes:

(6) $$\sqrt{T}\lambda \alpha_{A}\to g\frac{1-\lambda_{ch}^{2}}{1+{\left(g\lambda_{ch}\right)}^{2}}\sqrt{T}\lambda \alpha_{A}$$

When the modulation variance on Alice’s side $V_{A}=0$, the variance of the thermal state will be equal to the variance at Bob’s side, where ϵ is the excess noise of the CV-MDI-QKD system channel. That is:

(7) $$\frac{1+\lambda_{ch}^{2}}{1-\lambda_{ch}^{2}}=1+T\epsilon \Rightarrow \lambda_{ch}^{2}=\frac{T\epsilon }{T\epsilon +2}$$

After the NLA’s amplification, the parameter $\lambda_{ch}$ will be changed to $g\lambda_{ch}$ and then:

(8) $$\lambda_{ch}^{2}=\frac{T\epsilon }{T\epsilon +2}\to {\lambda^{\prime }}_{ch}^{2}=g^{2}\frac{T\epsilon }{T\epsilon +2}$$

Finally, we consider the action of the NLA when Bob does not have any knowledge of Alice’s measurement result. In this case, Bob’s state is regarded as a thermal state $\hat{\rho }\left(\lambda_{d}\right)=\left(1-\lambda_{d}^{2}\right)\sum {}_{n=0}^{\infty }\lambda_{d}^{2n}|n\rangle \langle n|$, where the variance is:

(9) $$V\left(\lambda_{d}\right)=\frac{1+\lambda_{d}^{2}}{1-\lambda_{d}^{2}}=TV_{A}+T\epsilon +1$$

According to Equation (9) and based on the analysis above, we know that the parameter $\lambda_{d}$ will change to $g\lambda_{d}$ after NLA, and the following equation will be obtained:

(10) $$\lambda_{d}^{2}=\frac{\lambda^{2}T\left(2-\epsilon \right)+T\epsilon }{2-2\lambda^{2}\left(1-T\right)+T\epsilon \left(1-\lambda^{2}\right)}\to {\lambda^{\prime }}_{d}^{2}=g^{2}\frac{\lambda^{2}T\left(2-\epsilon \right)+T\epsilon }{2-2\lambda^{2}\left(1-T\right)+T\epsilon \left(1-\lambda^{2}\right)}$$

By Equations (8) and (10), we can obtain:

(11) $$\begin{array}{c} T_{d}=\frac{4Tg^{2}}{{\left[T\epsilon \left(1-g^{2}\right)+2\right]}^{2}}\\ \epsilon_{d}=\epsilon -\frac{1}{2}T\left(g^{2}-1\right)\epsilon^{2} \end{array}$$

When the parameters of the CV-MDI-QKD system λ,T, and ϵ are replaced by the improved channel equivalent parameter $\lambda_{d},T_{d}$, and $\epsilon_{d}$, we can find that the signal-to-noise ratio of system will be improved. There are also some limitations we should pay attention to in the developed method provided above. When we calculate the secret key rate after the equivalent channel, the equivalent parameters must satisfy the following relationship: 0 $<\lambda_{d}$ < 1, 0 ≤ $T_{d}$ < 1, $\epsilon_{d}\geq$ 0. We put this restriction into Equation (11). Then, we can obtain the following restriction:

(12) $$g_{max}\left(\lambda ,T,\epsilon \right)=Min\left(\sqrt{1+\frac{1-\lambda }{T\lambda^{2}}},\frac{-1+\sqrt{4+4\epsilon (2+T\epsilon )}}{\sqrt{T}\epsilon }\right)$$

**Figure 3:**
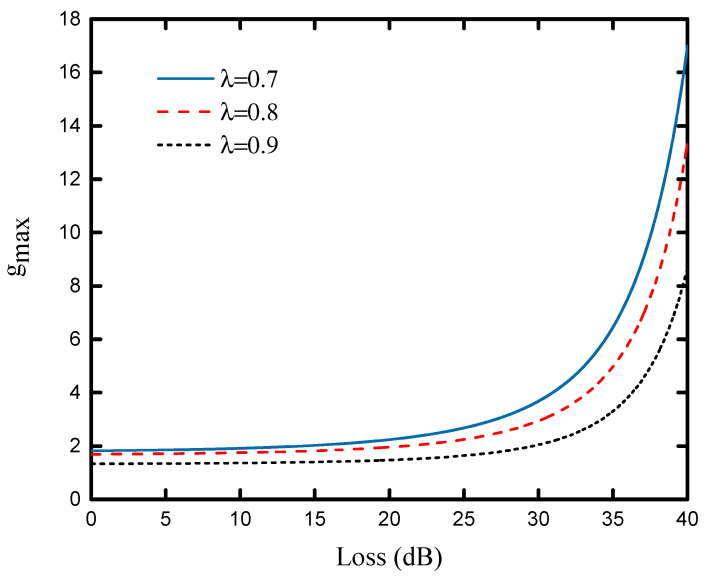
The maximum value $g_{max}$ of NLA gain is a function of channel loss (dB). The curve from top to bottom is λ=0.7,0.8,0.9. The excess noise is $\epsilon_{A}=\epsilon_{B}=0.001$.

According to Equation (12), we can obtain an upper bound of the parameter *g* of the NLA. As shown in Figure 3, the maximum value $g_{max}$ of NLA with channel loss is plotted under different values of the entanglement coefficient, i.e., λ=0.7,0.8,0.9. The result shows that the gain *g* of the NLA is limited to a small rang at the short communication distance, but when the communication distance is long, the gain of the NLA will increase quickly. In addition, the smaller the entanglement coefficient is, the greater the gain will be under the same communication distance.

## 3. The Secret Key Rate of the CV-MDI-QKD System with NLA

In the previous chapter, we introduced the amplification performance of NLA in detail, the equivalent parameters of channel transmittance and excess noise of the CV-MDI-QKD system with NLA are obtained. Then, we will calculate the secret key rate of the CV-MDI-QKD system with NLA, and the performance of the system will be simulated.

Assuming that the quantum channel parameters transmittance between Alice (Bob) and Charlie are $T_{A}=10^{-aL_{AC}/10}$ ($T_{B}=10^{-aL_{BC}/10}$), here, the quantum channel losses are a=0.2 dB/km. The excess noise is $\epsilon_{A}\left(\epsilon_{B}\right)$ correspondingly. After performing the quantum channel equivalent, the covariance matrix of $\rho_{A_{1}B_{1}^{\prime }}^{NLA}$ has the following form:

(13) $$\gamma_{A_{1}B_{1}^{\prime }}=\left[\begin{array}{cc} aII_{2} & c\sigma_{z}\\ c\sigma_{z} & bII_{2} \end{array}\right]=\left[\begin{array}{cc} V\left(\lambda_{d}\right)II_{2} & \sqrt{T_{d}\left[{\left(V\left(\lambda_{d}\right)\right]}^{2}-1\right)}\sigma_{z}\\ \sqrt{T_{d}\left[{\left(V\left(\lambda_{d}\right)\right]}^{2}-1\right)}\sigma_{z} & T_{d}\left(V\left(\lambda_{d}\right)+\chi_{tot}\right)II_{2} \end{array}\right]$$

where $II_{2}$ is the 2 × 2 identity matrix and $\sigma_{z}$ is the Pauli matrix $\sigma_{z}=\left[\begin{array}{cc} 1 & 0\\ 0 & -1 \end{array}\right]$. $\chi_{tot}$ represents the total noise at the input of the channel, $\chi_{tot}=\chi_{line}+\frac{2\chi_{hom}}{T}$, $\chi_{hom}$ represents the noise of the homodyne detector, $\chi_{hom}=\frac{1+v_{el}}{\eta }-1$, $\chi_{line}$ represents the noise of the quantum channel, and $\chi_{line}=\frac{1}{T}-1-\epsilon^{\prime }$. Here, $\epsilon^{\prime }$ refers to the equivalent excess noise of the equivalent one-way protocol, which can be calculated by:

(14) $$\begin{array}{c} \epsilon^{\prime }=1+\chi_{A}+\frac{T_{B}\left(\chi_{B}-1\right)}{T_{A}}+\frac{1}{T_{A}}{\left(\frac{\sqrt{2}}{k}\sqrt{V_{B}-1}-\sqrt{T_{B}}\sqrt{V_{B}+1}\right)}^{2} \end{array}$$

where $\chi_{A}=\epsilon_{A}-1+\frac{1}{T_{A}},\chi_{B}=\epsilon_{B}-1+\frac{1}{T_{B}}$ and when we set $k=\sqrt{\frac{2(V_{B}-1)}{T_{B}\left(V_{B}+1\right)}}$, we will obtain:

(15) $$\begin{array}{c} \epsilon^{\prime }=\epsilon_{A}+\frac{1}{T_{A}}\left[2+T_{B}\left(\epsilon_{B}-2\right)\right] \end{array}$$

Considering collective attack, the secret key rate of the CV-MDI-QKD protocol with NLA under finite-size effect can be defined as follows [46]:

(16) $$K_{finite}=P_{nla}\left[\frac{n}{N}\left(\beta I_{AB}-\chi_{BE}-\Delta \left(n\right)\right)\right]$$

where $P_{nla}$ is the probability of successful amplification of the NLA and $P_{nla}=1/g^{2}$, *N* is the length of valid data collected, *n* is the data length used for the final key rate generation, m=N−n is the data length for parameter estimation, β is the efficiency of reverse reconciliation, Δ(n) is a function related to privacy enhancement, and $\Delta \left(n\right)=7\sqrt{\frac{\log_{2}\left(2/\bar{\epsilon }\right)}{n}}$, where $\bar{\epsilon }$ means the smoothing parameter.

Moreover, $I_{AB}$ is the Shannon mutual information between Alice and Bob, which can be written as:

(17) $$I_{AB}=2\times \frac{1}{2}\log_{2}\frac{V_{A}}{V_{A|B}}=\log_{2}\frac{a+1}{a+1-c^{2}/\left(b+1\right)}$$

where $V=V_{A}+1$. From Equation (9), we can obtain the modulation variance $V_{A}=\frac{V(\lambda )-1-T\epsilon }{T}$, where λ is substituted with the equivalent $\lambda_{d}$.

In addition, the maximum information $\chi_{BE}$ that Eve can eavesdrop from Bob is limited by the Holevo quantity:

(18) $$\chi_{BE}=S\left(\rho_{E}\right)-\int dm_{B}p\left(m_{B}\right)S\left(\rho_{E}^{m_{B}}\right)$$

where $m_{B}$ represents Bob’s measurement results, $p(m_{B})$ represents the measured probability density, $\rho_{E}^{m_{B}}$ represents Eve’s state under Bob’s measurement, and S(ρ) represents the von Neumann entropy of the quantum state ρ. Since Eve can purify the system $\rho_{A_{1}B_{1}^{\prime }}$, we can obtain $S\left(\rho_{E}\right)=S\left(\rho_{A_{1}B_{1}^{\prime }}\right)$. Therefore, $\chi_{BE}$ can be expressed as:

(19) $$\chi_{BE}=S\left(\rho_{A_{1}B_{1}^{\prime }}\right)-S\left(\rho_{A_{1}}^{m_{B_{1}^{\prime }}}\right)$$

where $\rho_{A_{1}B_{1}^{\prime }}$ and $\rho_{A_{1}}^{m_{B_{1}^{\prime }}}$ are the covariance matrices of $\gamma_{A_{1}B_{1}^{\prime }}$ and $\gamma_{A_{1}}^{m_{B_{1}^{\prime }}}$, respectively. $S(\rho_{A_{1}B_{1}^{\prime }})$ is the function of the symplectic eigenvalues $\lambda_{1,2}$ of $\gamma_{A_{1}B_{1}^{\prime }}$, which can be expressed as:

(20) $$\begin{array}{c} S\left(\rho_{A_{1}B_{1}^{\prime }}\right)=G\left(\frac{\lambda_{1}-1}{2}\right)+G\left(\frac{\lambda_{2}-1}{2}\right) \end{array}$$

where $G\left(x\right)=\left(x+1\right)\log_{2}\left(x+1\right)-x\log_{2}x$ and the symplectic eigenvalues $\lambda_{1,2}$ is:

(21) $$\begin{array}{c} \lambda_{1,2}^{2}=\frac{1}{2}\left[A\pm \sqrt{A^{2}-4B}\right] \end{array}$$

and

(22) $$\begin{array}{cc} \; & A=a^{2}+b^{2}-2c^{2}\\ \; & B={\left(ab-c^{2}\right)}^{2} \end{array}$$

Moreover, $S(\rho_{A_{1}}^{m_{B_{1}^{\prime }}})$ is the function of the symplectic eigenvalues $\lambda_{3}$ of $\gamma_{A_{1}}^{m_{B_{1}^{\prime }}}$, which will be given by:

(23) $$\begin{array}{c} S\left(\rho_{A_{1}}^{m_{B_{1}^{\prime }}}\right)=G\left(\frac{\lambda_{3}-1}{2}\right) \end{array}$$

the symplectic eigenvalues $\lambda_{3}$ is

(24) $$\begin{array}{c} \lambda_{3}=a-\frac{c^{2}}{b+1} \end{array}$$

**Figure 4:**
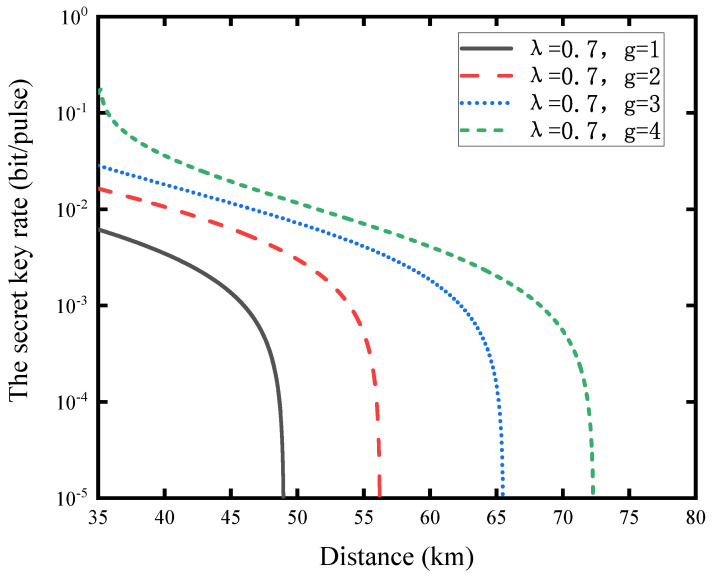
The secret key rate as a function of the communication distance. The entanglement coefficient λ=0.7 and *g* is the gain of NLA. The other parameters are $\epsilon_{A}=\epsilon_{B}=0.002$, $v_{el}=0,\eta =1$, and β=0.98.

To demonstrate the influence of the NLA on the secret key rate of the CV-MDI-QKD system, we simulate the relationship between the secret key rate of the system and the communication distance under different NLA gains in Figure 4. Here, we choose the optimal entanglement coefficient λ=0.7 and consider that Charlie’s detectors are perfect, that is, $\chi_{tot}=\chi_{line}$. Among all curves, the black curve with gain g=1 represents the secret key rate of the original CV-MDI-QKD protocol. We can see that its performance is worse than the CV-MDI-QKD protocol with NLA in the same communication distance. Moreover, the result shows that after adding NLA to the CV-MDI-QKD system, the communication distance is longer than that of the original protocol. Furthermore, the communication distance and the secret key rate of the system will increase significantly with the increase in the NLA’ s gain.

**Figure 5:**
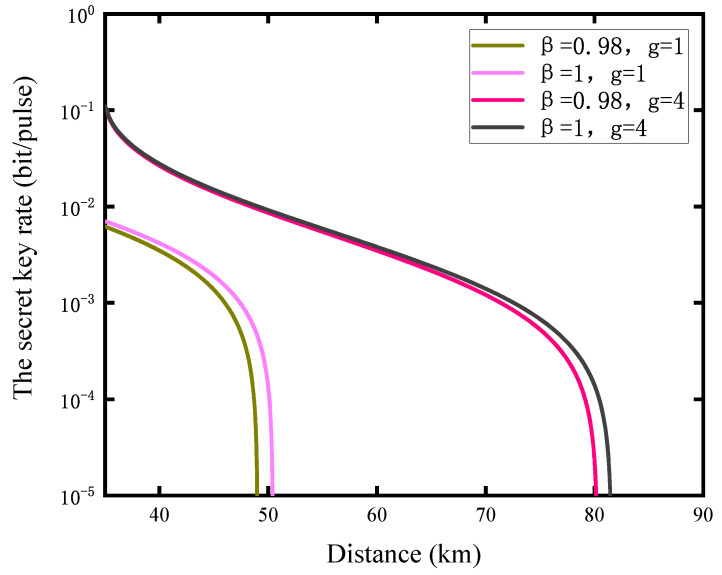
The secret key rate is a function of communication distance under different the efficiencies of reverse reconciliation and the gain of NLA. In the NLA-based CV-MDI-QKD protocol, when g=4, the reverse reconciliation efficiency has little effect on the secret key rate of the system. The other parameters are $\lambda =0.7,\epsilon_{A}=\epsilon_{B}=0.002,$ and $v_{el}=0,\eta =1.$

Moreover, the effect of the reverse reconciliation efficiency on the system is shown in Figure 5. We plot the relationship between the secret key rate and the communication distance with different reverse reconciliation efficiency and the NLA’s gain. The result shows that the influence of the reverse reconciliation efficiency on the communication distance is relatively small when the gain of the NLA becomes larger. In short-distance communication, the greater the gain of NLA, the smaller the impact of the reverse reconciliation efficiency on the secret key rate.

**Figure 6:**
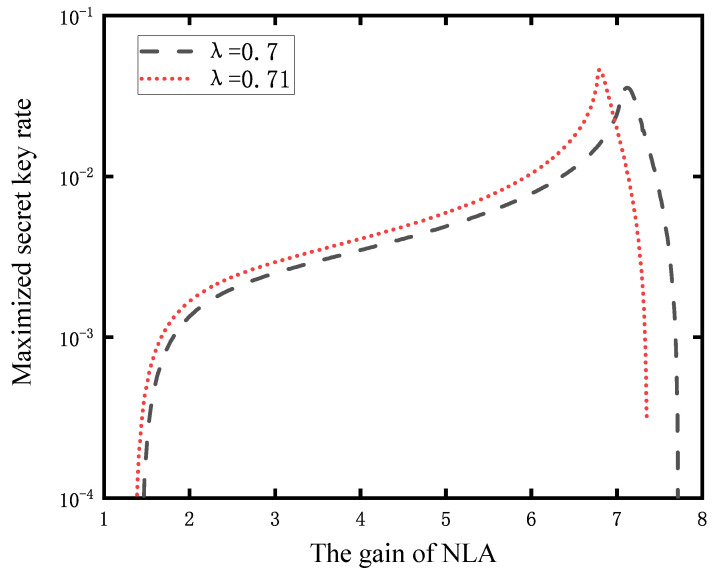
Maximized secret key rate as a function of the gain of NLA, with a probability of success $P_{nla}=1/g^{2}$. The black dotted curve represent the secret key rate of the NLA-based CV-MDI-QKD protocol with λ=0.7, and the red curve represents the secret key rate of the NLA-based CV-MDI-QKD protocol with λ=0.71.

Finally, to investigate the best gain to maximize the secret key rate of the CV-MDI-QKD system with NLA, we simulate the maximized secret key rate as a function of the gain of NLA. Assuming that the secure communication distance of the CV-MDI-QKD system is 60 km, we can see that the secret key rate of the system does not increase with the increase in the NLA’s gain but has a maximum in Figure 6. The reason for this is that there is a successful amplification probability of NLA is $P_{nla}=1/g^{2}$. With the increase in the NLA’s gain, the secret key rate will reach the optimal value with a certain gain, and then the secret key rate will drop rapidly. The best gain under different modulation variances is also given by Figure 6, and it can be seen that the smaller the modulation variance, the greater the optimal gain required.

## 4. Conclusions

In this paper, we induced an NLA into the traditional CV-MDI-QKD protocol to improve the communication distance and the secret key rate of the system. The NLA is added to the output of the detector at Charlie’s side first, and then, to investigate the performance of the system with NLA, we equate the E-B model of the CV-MDI-QKD system with NLA to the one-way CV-QKD protocol for which both Alice and Bob use heterodyne detectors and perform some related simulations. The research results show that NLA can increase the communication distance and secret key rate of the CV-MDI-QKD protocol. However, the secret key rate will reach the optimal value with a certain gain, and then the secret key rate will drop rapidly with the increase in the NLA’s gain since there is a successful amplification probability of NLA. Moreover, we find that the influence of the reverse reconciliation efficiency on the communication distance is relatively small when the gain of the NLA becomes larger.

## Data Availability

Data underlying the results presented in this paper are not publicly available at this time but may be obtained from the authors upon reasonable request.
